# Size-dependent phase diagrams of metallic alloys: A Monte Carlo simulation study on order–disorder transitions in Pt–Rh nanoparticles

**DOI:** 10.3762/bjnano.3.1

**Published:** 2012-01-02

**Authors:** Johan Pohl, Christian Stahl, Karsten Albe

**Affiliations:** 1 Institut für Materialwissenschaft, Technische Universität Darmstadt, Petersenstr. 32, D-64287 Darmstadt, Germany

**Keywords:** Monte Carlo simulation, nanoparticles, nanothermodynamics, phase diagram, Pt-Rh, thermodynamics

## Abstract

Nanoparticles of Pt–Rh were studied by means of lattice-based Monte Carlo simulations with respect to the stability of ordered *D*0_22_- and 40-phases as a function of particle size and composition. By thermodynamic integration in the semi-grand canonical ensemble, phase diagrams for particles with a diameter of 7.8 nm, 4.3 nm and 3.1 nm were obtained. Size-dependent trends such as the lowering of the critical ordering temperature, the broadening of the compositional stability range of the ordered phases, and the narrowing of the two-phase regions were observed and discussed in the context of complete size-dependent nanoparticle phase diagrams. In addition, an ordered surface phase emerges at low temperatures and low platinum concentration. A decrease of platinum surface segregation with increasing global platinum concentration was observed, when a second, ordered phase is formed inside the core of the particle. The order–disorder transitions were analyzed in terms of the Warren–Cowley short-range order parameters. Concentration-averaged short-range order parameters were used to remove the surface segregation bias of the conventional short-range order parameters. Using this procedure, it was shown that the short-range order in the particles at high temperatures is bulk-like.

## Introduction

**Figure 1:**
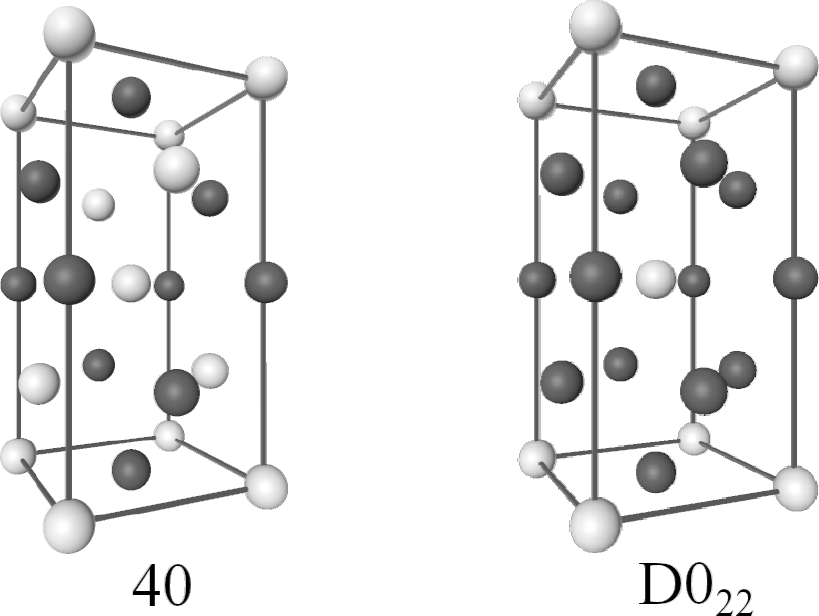
The structure of the ordered phases with Strukturbericht designation “D0_22_” (prototype structure: Al_3_Ti, space group: *I*4/*mmm*, No. 139) and “40” (space group: *I*4_1_/*amd*, No. 141). “40” is an unconventional designation, which has been used in the context of studies on the Pt–Rh alloy since the prediction of the phase in [3] as no Strukturbericht designation or prototype exists. The structure was identified in 1971, without a specific name being assigned, as an antiferromagnetic ground state of the Ising model with certain ratios of the first- and second-nearest-neighbor interactions on a face-centered cubic lattice [29].

Pt–Rh is an important alloy due to its catalytic activity in different reactions. In the past it was assumed that Pt–Rh is immiscible at low temperatures [1–2], but theoretical studies revealed that Pt–Rh forms the intermetallic phases 40 and *D*0_22_ [3], which are thermodynamically stable below room temperature according to a recently published theoretical phase diagram [4] (see Figure 1 for the 40 and *D*0_22_ structures).

Boundaries in a bulk phase diagram are well defined in the case of the thermodynamic limit (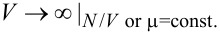), where first-order phase transitions are indicated by singularities in the grand canonical potential [5]. In the case of finite systems, however, the thermodynamic limit cannot be used, and as a consequence there are no sharp first-order phase transitions in the canonical and the grand canonical ensemble [6] and therefore different theories are needed in order to describe small systems. The thermodynamics of small systems has been developed by Hill as early as 1963 [6], but recently regained interest due to its applicability in modern nanoscience and engineering. The theory was recently redeveloped on a more intuitive foundation [7] under the term “nanothermodynamics” [8] by the same author. Recent microcanonical approaches pioneered by Gross et al. [9–10] explore the topology of the entropy surface *S*(*E,N_i_*) as a function of the energy *E* and the particle number *N*. Studying the entropy surface in the microcanonical ensemble can be a useful theoretical tool, because convex intruders of the entropy can be used as a concept to assign first-order transitions even in finite systems, and additional quantities, such as the interface tension between phases, become accessible [9]. A quantitative assessment of the entropy surface is in principle possible by the Wang–Landau algorithm [11] but a tedious task for a more complex Hamiltonian. Furthermore, the choice of the microcanonical ensemble corresponds to a completely isolated system, which does not exchange energy with its environment. Such conditions are experimentally possible [10], but hard to realize and clearly not satisfied for the case of nanoparticles in equilibrium with their substrate.

Phase equilibria between solid and liquid phases in binary alloy particles were, for example, investigated for Cu–Ni [12], Sn–Bi [13], Pb–Bi [14] and other eutectics [15–17], as well as for miscible alloys [18–20]. In addition, there are some studies related to the phase diagrams of solid–solid phase equilibria of alloys with a miscibility gap [21–23]. Such a case has also recently been experimentally studied for the case of Au–Pt [23]. More general thermodynamic treatments of phase separation in nanoparticles were given by Wautelet et al. [22] and Norskov et al. [21]. The latter authors focused especially on phase equilibria of immiscible Ag–Cu nanoparticles by means of Monte Carlo simulations and found that for all studied alloys phase separation becomes impossible below a certain critical size at any temperature [21]. Significantly fewer studies are found on ordering nanoalloys. Recently, a study on the equilibrium ordering properties of Au–Pd bulk and nanoalloys was published by Atanasov and Hou [24]. Ordering Fe–Pt nanoalloys were studied by means of lattice Monte Carlo simulations [25].

However, as far as we know, a complete size-dependent phase diagram of an ordering nanoalloy has not yet been studied. It is our intention to examine such a size-dependent phase diagram by using the model system of Pt–Rh particles and thereby extending our previous results for bulk Pt–Rh to the nano regime. We will first give a short review of the refined BOS mixing model. This model will then be parameterized for Pt–Rh particles. The resulting phase diagrams for three different particle sizes will finally be discussed. We address the question of how to interpret a two-phase equilibrium in the particle, and the effects related to surface segregation are examined. Furthermore, we evaluate the Warren–Cowley short-range order (WC-SRO) parameters in order to investigate the order–disorder phase transitions in the particles. These parameters prove to be useful in order to assign a critical temperature to the smooth phase transitions in this finite system.

## Method

### The refined BOS mixing model

We use a standard Metropolis Monte Carlo algorithm that implements a simulation model that is closely related to the “Bond Order Simulation” (BOS) Mixing model [26]. We have applied this model before in order to calculate the bulk phase diagram of Pt–Rh and to examine the ordering behavior [4].

In our refined version of the BOS mixing model the lattice energy is given by a sum over the site energies

[1]
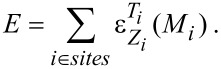


The site energies 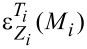 depend on the type *T_i_* of the atom, its total first-neighbor coordination *Z_i_* and its number of unequal first to fourth nearest neighbors *M_i_*. The site-energies are given by the following expressions in our model:

[2]
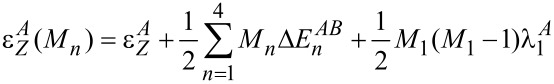


[3]
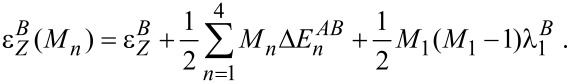


Here the 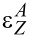 and 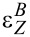 are on-site constants that bear the dependency on the total coordination *Z* of first nearest neighbors. The remaining 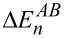 with *n* = 1, …, 4, 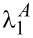 and 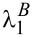 are used to describe the atomic interactions between different species of atoms. *M*_1_, …, *M*_4_ are the number of unequal first to fourth nearest neighbors for the corresponding atom. The sum terms are the linear interactions up to the fourth nearest neighbor, while the last terms in the site-energies are nonlinear terms, that only apply to first neighbors. Such nonlinear terms are necessary in order to model asymmetric phase diagrams. In the case of the Pt–Rh bulk phase diagram the asymmetry is reflected by the fact that the *D*0_22_-phase is present at a 1:3 stoichiometry of platinum to rhodium, while it is not at 3:1. The model assumes a rigid lattice and thus neglects possible relaxation effects. Please refer to [4] for a more detailed description of the model.

### Parameterization of the model

In order to apply our model to particles, we must parameterize the site-energy constants 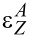 and 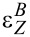. These constants are not needed for bulk calculations as they would only contribute a constant energy term to the total lattice energy. This is different in the case of a particle. The parameters needed for modeling the mixing and ordering behavior are taken from the bulk parameterization. These are the 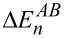 with *n* = 1, …, 4 and 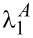, 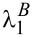 and their values are given in Table 1.

**Table 1:** The interaction mixing parameters used in our calculations in meV.

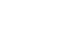	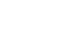	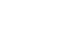	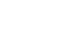	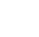	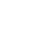

0.1487	1.4730	1.2192	−1.3731	−1.5533	−0.5907

We obtain the site-energy constants from the cohesive energies *E_coh_* = *ε*_12_ and the surface energies for the fcc(111) and fcc(100) surface σ_111_ = *ε*_12_ − *ε*_9_ and σ_100_ = *ε*_12_ − *ε*_8_. The used values for the surface energies given in Table 2 were taken from [27], in which they were obtained by the scalar relativistic full-potential screened Korringa–Kohn–Rostoker method (FP-KKR) and by using the local-density approximation (LDA). For the cohesive energies we assumed 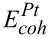 = 5.84 eV and 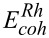 = 5.75 eV. In order to calculate the configuration of a Wulff-shape particle for a face-centered cubic lattice, i.e., a truncated octahedron, we also need the site-energy constants for *Z* = 7 and *Z* = 6. However, for future applications to simulations of particles with varying shape it seems appropriate to determine the site-energy constants for all *Z* directly. Therefore, we determine the site-energy constants for all *Z*, other than 12, 9 or 8, by fitting a site-energy function with a square-root dependence on the coordination to the three values we already have. In addition we demand that 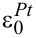 = 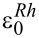 = 0. All the determined site-energy constants are given in Table 3.

**Table 2:** Surface energies of platinum and rhodium taken from [27] (FP-KKR).

element	surface	energy (eV/atom)	energy (*J*/*m*^2^)

Pt	(111)	0.957	2.31
Pt	(100)	1.272	2.65
Rh	(111)	1.034	2.65
Rh	(100)	1.404	3.12

**Table 3:** The site energy constants 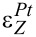 and 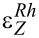 in eV.

*Z*	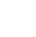 (eV)	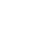 (eV)

0	0.0	0.0
1	−1.119	−0.963
2	−1.722	−1.532
3	−2.241	−2.036
4	−2.716	−2.506
5	−3.162	−2.955
6	−3.588	−3.346
7	−3.867	−3.708
8	−4.568	−4.346
9	−4.883	−4.716
10	−5.171	−5.029
11	−5.546	−5.423
12	−5.840	−5.750

## Simulation

We have calculated a bulk phase diagram including configurational entropy by thermodynamic integration in the semi-grand canonical ensemble before [4]. The approach was formerly proposed by van de Walle et al. [28], but applied within the framework of the cluster-expansion formalism. The cluster-expansion formalism is not easily applicable in the vicinity of a surface or an edge of a particle due to the lack of symmetry, although there are approaches that also include “surface figures” in the cluster expansion. The model we introduced is able to accurately calculate the equilibrium configuration of binary-alloy particles. It features full control over the mixing properties and surface-energy differences of the pure elements, which is the driving force for surface segregation in our model. The dependency of the phase diagram on particle size is examined on the basis of three nanometer-sized particles with *n* = 9201, 2075 and 807 atoms, corresponding to a diameter of 7.8, 4.3 and 3.1 nm, respectively. The cluster shape was obtained from a face-centered cubic lattice by the Wulff construction by using the (100) and (111) surface energies for platinum and rhodium. The cluster shape was fixed during the simulations. The ratios between the (100) and the (111) surface energies are very similar for platinum and rhodium (Pt: 1.33, Rh: 1.36), thus resulting in the same Wulff-shape for the particle sizes we used. At low temperature it is therefore justified to constrain the particle shape to the Wulff-shape even when varying composition. From the initial cluster shapes with a random alloy configuration we performed thermodynamic integration in the semi-grand canonical ensemble. This ensemble fixes the total number of atoms *N*, but not the individual number of atoms *N_Pt_* and *N_Rh_*. The difference in the chemical potentials Δμ = μ*_Pt_* − μ*_Rh_* is used as a control variable in addition to the temperature *T*. The method allows the calculation of the total free-energy surface as a function of temperature and composition. Although there is not a simple equation for the high-temperature expansion of the free energy in the case of particles, we are still able to perform thermodynamic integration to obtain free-energy differences. The integration is performed from *T* = 285 K down to *T* = 50 K in 5 K steps. For each temperature step the system was equilibrated for 900 MC steps and then data was averaged over 100 MC steps, taking five data points per MC step by using a simple Metropolis Monte Carlo algorithm. In the semi-grand canonical ensemble a trial step consists of a single spin flip, i.e., changing a Pt atom to a Rh atom, or vice versa.

**Figure 2:**
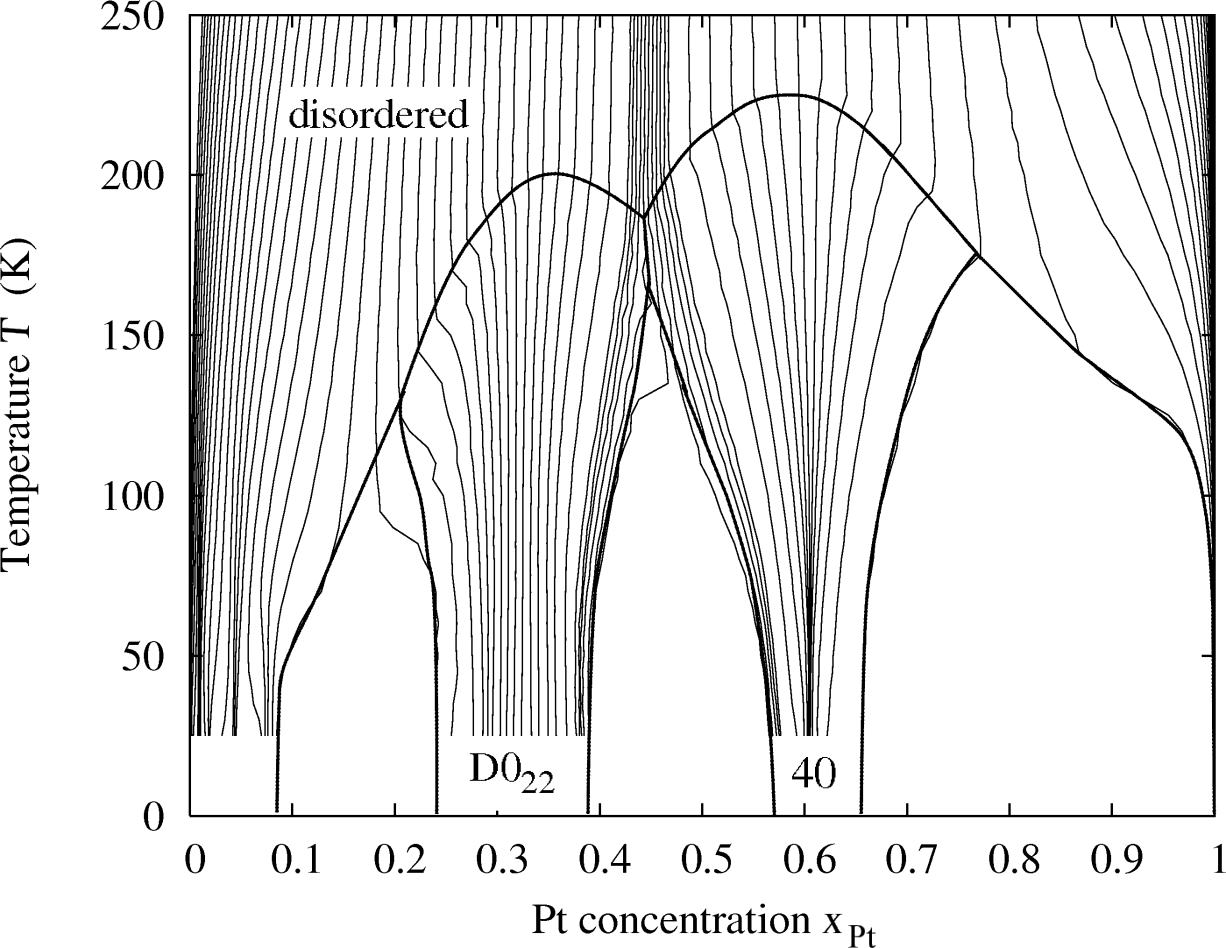
The vertical lines in the plot represent equipotential lines of the free energy for constant difference in the chemical potential between the two species Pt and Rh. These were obtained by thermodynamic integration in the semi-grand canonical ensemble. The phase diagram for the large particle (9201 atoms, *d* = 7.8 nm) was constructed from these lines and the order parameters, and is represented by the thicker solid line.

**Figure 3:**
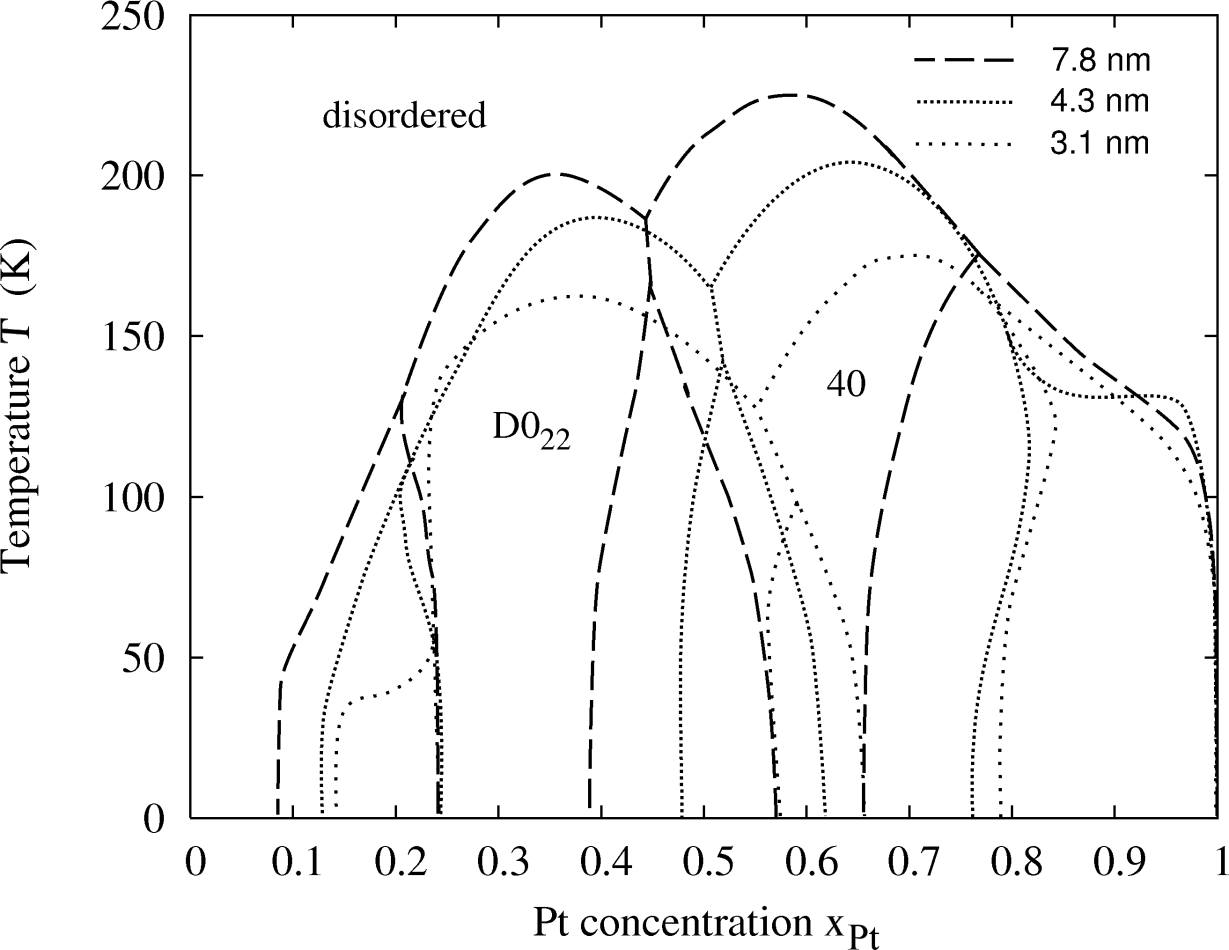
Calculated phase diagrams of Pt–Rh for three different particle sizes of 9201, 2075 and 807 atoms, corresponding to diameters of 7.8, 4.3 and 3.1 nm.

**Figure 4:**
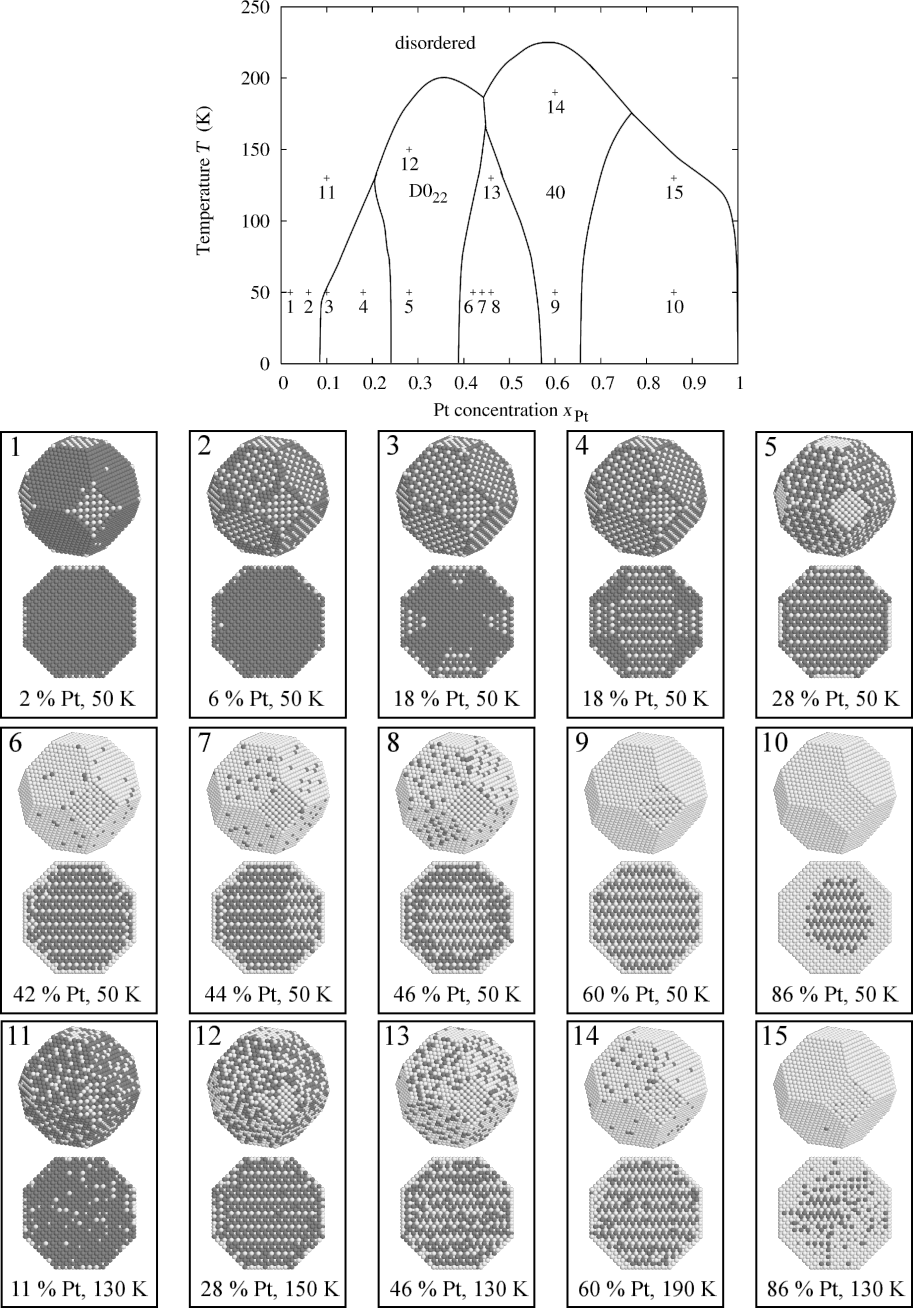
Equilibrium configurations of large particles with 9201 atoms at different concentrations and temperatures and their position in the nanoparticle phase diagram. The numbers in the phase diagram correspond to the particle pictures below it.

The lines that are finally obtained in the plot of temperature against concentration (Figure 2) are equipotential lines for which the difference between the chemical potentials Δμ = μ*_Pt_* − μ*_Rh_* of the two constituents is constant. These lines do not cross the two-phase regions, and therefore the line close to a void region can be taken as a phase boundary. In addition we evaluated the Warren–Cowley order parameters to map out the phase boundary between the disordered phase and the ordered phases. The three phase diagrams for different particle sizes are shown in Figure 3. In order to validate the obtained phase diagrams and to obtain atomic structure data in the two-phase regions, which are inaccessible in the semi-grand canonical ensemble, we also performed simulations in the canonical ensemble. Some representative particle configurations are shown in Figure 4. However, the low-temperature ordered phases of Pt–Rh are probably not observable in experiment due to the fact that vacancy diffusion is frozen at these temperatures, making it impossible to establish thermodynamic equilibrium in the particle within an observable time frame. Nevertheless, the results should give us valuable general insights into ordered-phase equilibria in nanoparticles.

In the following, Warren–Cowley short-range order parameters (WC-SRO) [30] are used for the analysis of short-range order in the bulk and in particles. The WC-SRO are originally defined as

[4]
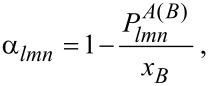


where 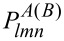 is the probability to find a B atom as a neighbor of a given A atom, and *x_B_* is the concentration of B atoms. It can be shown that in the case of a bulk material it does not matter whether the parameter is centered at an A or a B atom, i.e.,

[5]
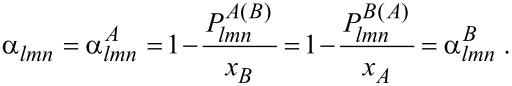


However, for finite systems with a surface, generally 
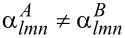
, as was discussed by Atasanov and Hou recently [31]. They also suggested the use of a concentration-averaged short-range order parameter

[6]
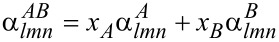


as a more suitable measure for the order in the core of the particles. In this paper, we use both definitions of the order parameters in order to obtain the maximum amount of information about the system. Another approach to probe ordering transitions in particles is to use the conventional ordering parameter as in Equation 5, but to restrict the calculation of the parameters to a small core region of the particle. This approach was also applied to validate our findings (see below in Figure 10). It has the advantage that the parameters converge to the bulk values of the corresponding ordered phases at low temperature; however, it also has the drawback that higher-order parameters cannot be calculated anymore, the statistics become weaker, and it may not be comparable to experimental data, to which surface atoms contribute.

## Results and Discussion

### Nanoparticle phase diagrams

**Figure 5:**
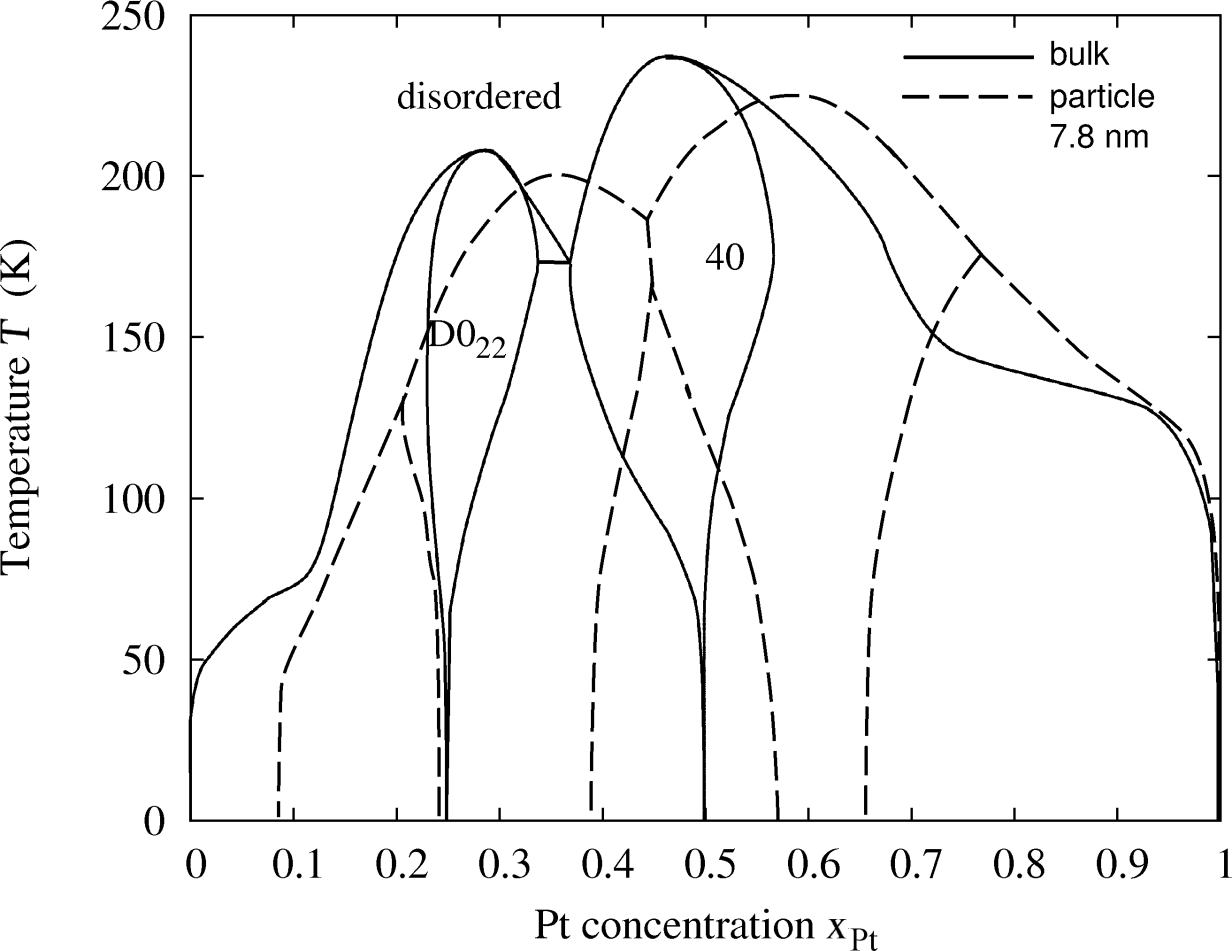
Calculated phase diagram of bulk Pt–Rh (solid line) in comparison to that of a particle consisting of 9201 atoms, 7.8 nm diameter (dashed line).

The calculated phase diagrams for nanoparticles show some features that are different from those in the bulk. These features may be explained in terms of the existence of surfaces and interfaces, and by finite-size effects. The interplay of surface segregation, surface ordering and bulk ordering is crucial. In order to explain the different phase-diagram features, it is instructive to compare the phase diagram for the bulk and for different particle sizes (Figure 5 and Figure 3) and to have a look at some particle configurations at a low temperature, as shown in Figure 4 for *T* = 50 K (see Figure 1 for the structure of the ordered phases *D*0_22_ and 40). The phase diagram for a particle of 9201 atoms with a diameter of 7.8 nm is compared to that of the bulk alloy in Figure 5. The ordering temperature in the case of a large particle consisting of 9201 atoms shifts down from the bulk temperature. The bulk ordering temperature of bulk Pt–Rh was calculated to be 238 K at 1:1 stoichiometry for the 40-structure and 209 K at 25 atom % Pt for the *D*0_22_-structure, whereas the maximum ordering temperatures for 7.8 nm particles are 198 K at 35 atom % Pt concentration for the *D*0_22_-phase and 226 K at 59.5 atom % Pt for the 40-phase. This trend towards lower ordering temperatures continues at smaller particle sizes, which can be identified from the particle phase diagrams in Figure 3 as well as from the short-range order parameters in Figure 8, Figure 9 and Figure 10 (see below).

The platinum solubility in a rhodium particle at low temperatures is much larger than that in the bulk. The solubility does not go to zero at zero temperature, but it hits the zero-temperature line at about 8 atom % platinum concentration. However, the term “solubility” has to be interpreted differently for a particle. For platinum concentration below 8 atom % at temperatures as low as 50 K, the excess platinum atoms do not dissolve inside the rhodium but they tend to stay at the surface and form a completely ordered surface layer (Figure 4, particles 1 and 2). This is a consequence of the preferred segregation of platinum atoms. The reason for segregation in our model is the difference between the surface energies of platinum and rhodium. Thus, we have to associate the shift in the phase boundary between the pure phase and the two-phase region with the emergence of a surface phase and surface solubility for the particle, not with an increase in the volume solubility. When the platinum concentration is increased upward from zero, platinum atoms tend to form ordered domains at the (100) facets first (Figure 4, particle 1), because the difference in surface energy is larger for the (100) surface as compared to the (111) surface. We find some interesting features for the two-phase equilibrium between the *D*0_22_- and the pure rhodium phase in the particle. In Figure 4, for particle 3, we see that pyramid-shaped ordered *D*0_22_-domains have formed below the (100) facets. The ordered surface layer of the (100) facet is compatible with *D*0_22_-ordering. This is not the case for the (111) facets, which is the reason why we see the domains form below the (100) facets and not below the (111) facets, under which we find pure rhodium. Additional energy would be needed to form a phase boundary with the top layer of a (111) facet and a *D*0_22_-domain, as the order of the facet is not compatible with the *D*0_22_-order. Interestingly, the configuration with the pyramids is energetically more stable than a core–shell two-phase equilibrium. At 18 atom % platinum concentration (Figure 4, particle 4) we see that one large *D*0_22_-domain has formed that connects two opposing (100) facets. Smaller *D*0_22_-domains formed below the remaining four (100) facets. There is a boundary between the small domains and the large domain as they are differently oriented. Both in the bulk and in the particle the formation of a fully ordered *D*0_22_ is possible from 25 atom % Pt concentration upwards. However, in the case of a particle the *D*0_22_-phase extends over a much broader concentration range. The trend continues when going to smaller particle sizes (see Figure 3). The reason for this is again the existence of the surface and its function as a reservoir for excess platinum atoms. The larger broadening of the *D*0_22_-region as well as the shrinking of the *D*0_22_/40 two-phase region for small particles can therefore be attributed to the fact that the surface reservoir for excess atoms is larger relative to the number of core atoms. We can see that, while the (100) facets already consist entirely of platinum atoms at 28 atom % total platinum concentration at a temperature of 50 K (Figure 4, particle 5), excess platinum atoms may still be incorporated into the (111) facets up to a concentration of about 42 atom % total platinum concentration. At this point the surface platinum concentration reaches a maximum. The two-phase region between the *D*0_22_- and the 40-phase reveals another interesting phenomenon: When the platinum concentration is increased by only 2 atom % from 42 to 44 atom % the platinum concentration at the surface drops while a two-phase equilibrium inside the particle is formed (Figure 4, particle 7). When the total platinum concentration is increased by another 2 atom % from 44 to 46 atom % the platinum surface concentration is lowered even more while the two-phase equilibrium inside the particle takes a core–shell shape. This is consistent with a small interface energy between the *D*0_22_- and the 40-phase of only 3.4 meV per interface atom, which has been estimated from the total energy of bulk simulations within the two-phase region. The behavior of a decrease in surface concentration, when the total concentration of platinum is increased, is therefore related to the increasing size of the 40-phase core. A larger core is energetically more favorable than a small one, even at the cost of drawing platinum atoms from the surface inside the particle to the 40-core. The formation energy of the 40-phase counter balances the driving force for surface segregation of platinum atoms, which is due to the surface energy of platinum being lower than that of rhodium. In reality, the size mismatch of platinum and rhodium may act as a another driving force for surface segregation. Size mismatch, however, is not accessible within a rigid lattice model. Above 46 atom % total platinum concentration the surface platinum concentration increases again. At 60 atom % platinum concentration we find a completely 40-ordered particle with a surface layer of platinum (Figure 4, particle 9). The consequence of having the top layer entirely filled with platinum is that the 40-phase does not broaden its concentration range when going to smaller particle sizes as much as the *D*0_22_-phase does (compare Figure 3). Between the 40-phase and the pure platinum phase the two-phase equilibrium is established as a core–shell configuration (Figure 4, particle 10). Going to temperatures above 50 K the configurational entropy starts to play an increasingly significant role. In Figure 4, for particle 11 at 130 K the ordered surface phase and subsurface ordering have vanished compared to the configuration at 50 K. In Figure 4, for particle 12 and 14, it shows that the *D*0_22_- and the 40-phase close to their maximally stable compositions incorporate defect atoms in the core, as is to be expected. The core of the two-phase core–shell equilibrium between the *D*0_22_- and the 40-phase becomes unstable and diffuse at 130 K (Figure 4, particle 13), but still both *D*0_22_- and 40-type ordering can be identified. In the two-phase region between the 40- and the pure platinum phase the core also becomes diffuse, but a smaller perfectly ordered 40-core can be identified (Figure 4, particle 15).

The size-dependent features, which are observed in the nanoparticle phase diagrams when the particle size decreases can be summarized as follows:

- The ordering temperatures of ordered phases in the particle shift downward.
- The global concentration stability ranges of the ordered phases broaden.
- The stable phases shift towards higher concentrations of the segregating element.
- The two-phase regions in the phase diagram shrink.
- An ordered surface phase emerges at low concentrations of the segregating element.

We conclude from the simulations that these features are likely to be found in ordering systems with a surface segregation tendency of one element.

### Short-range order and order–disorder transitions

**Figure 6:**
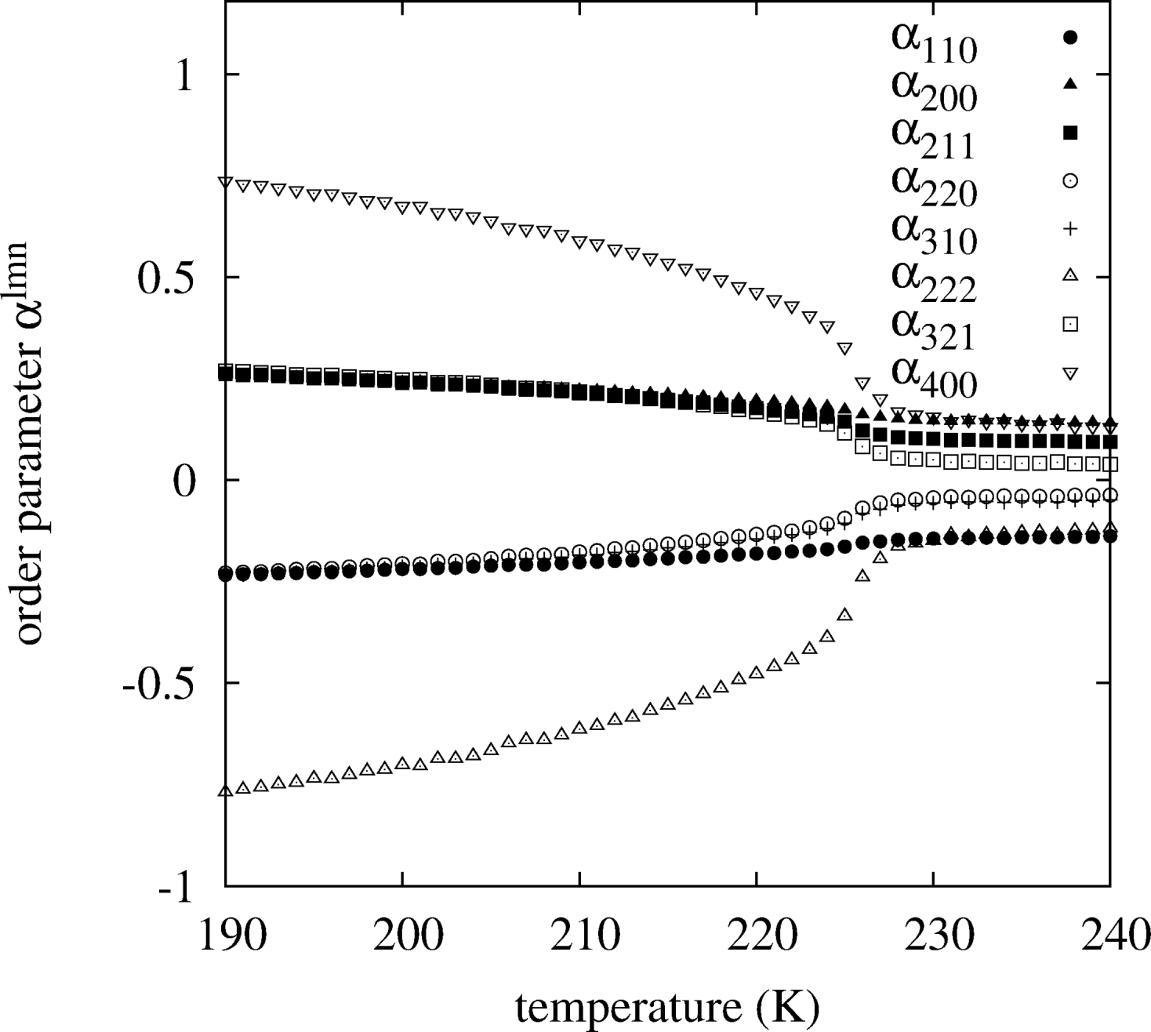
All concentration-averaged Warren–Cowley short-range order parameters 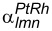 up to 8th neighbors for a Pt–Rh nanoparticle with 9201 atoms (corresponding to a diameter of 7.8 nm) in equilibrium versus temperature. The phase transition at *T* ≈ 226 K is clearly visible.

**Figure 7:**
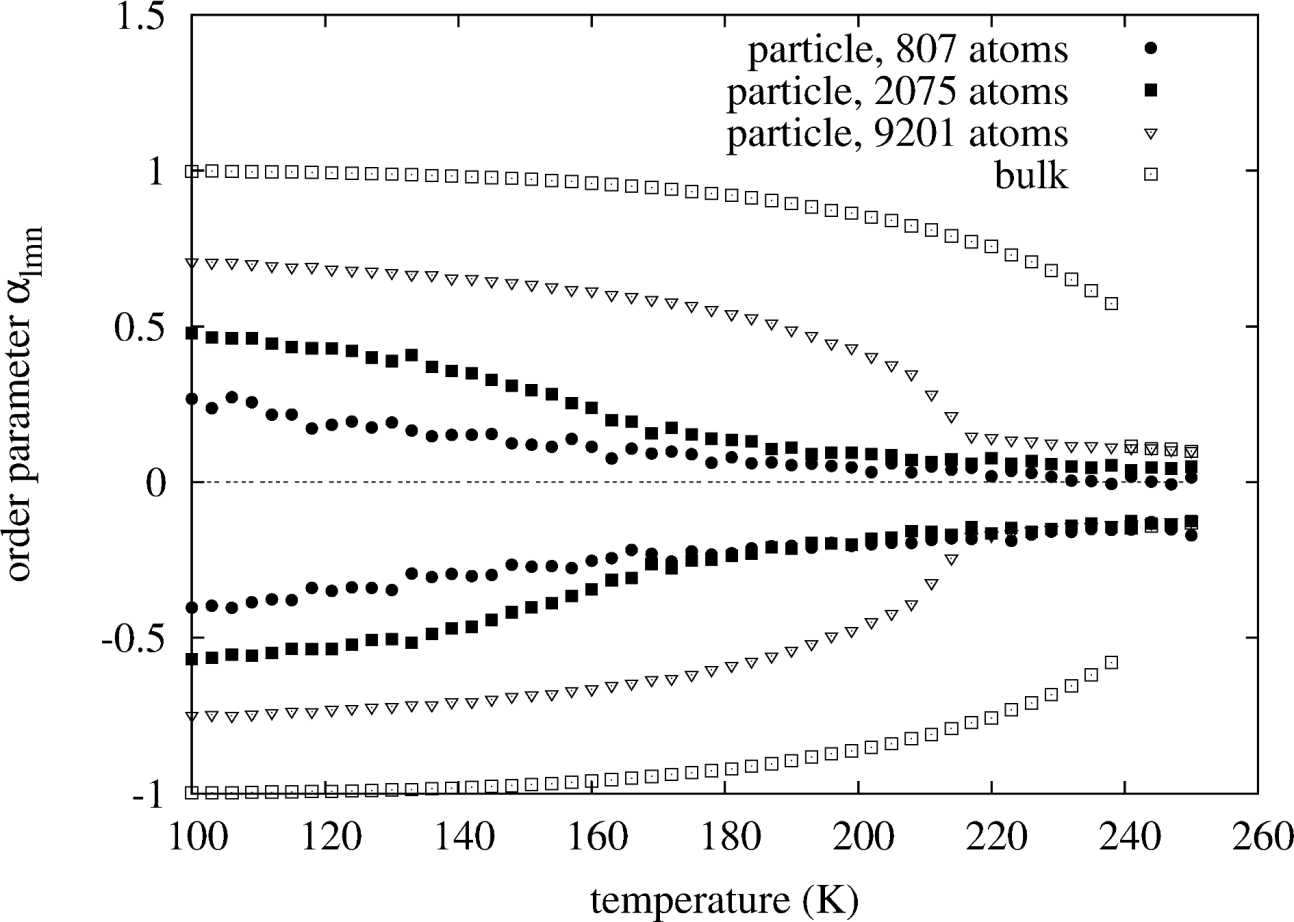
Comparison of the 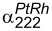 (lower points) and 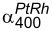 (upper points) WC-order parameters versus temperature for different particle sizes. Composition is fixed at 50 atom % Pt for all sizes. For the medium size and the small particle a transition cannot be identified.

**Figure 8:**
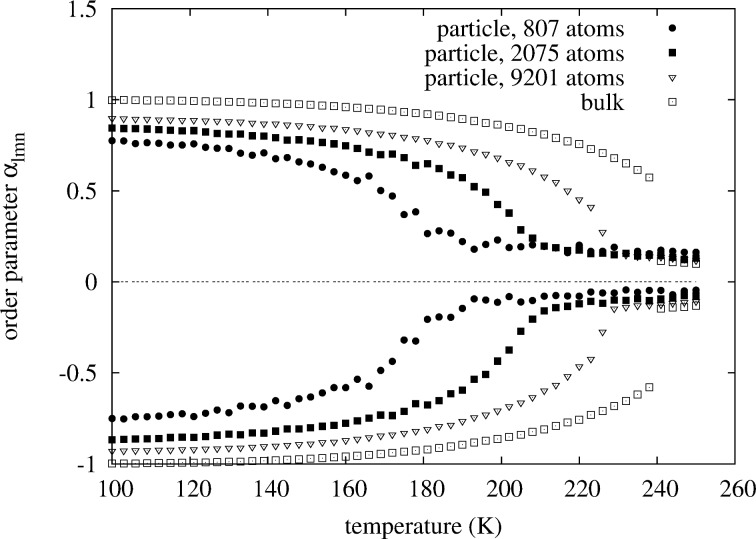
Comparison of the concentration-averaged order parameters 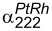 (lower points) and 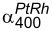 (upper points). The Pt concentration is chosen such that for all particles the phase transition is observed at the maximum critical temperature of the 40-phase. No shift in magnitude of the concentration-averaged parameters is seen as is the case for non-concentration-averaged 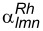 in Figure 9. Compare also to the core constrained short-range order parameters in Figure 10.

**Figure 9:**
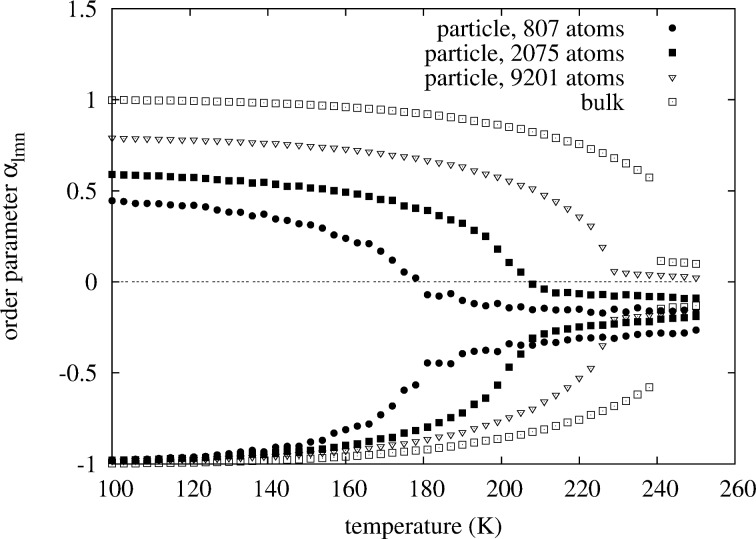
Comparison of the WC-order parameters 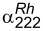 (lower points) and 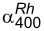 (upper points). The Pt concentration is chosen such that for all particles the phase transition is observed at the maximum critical temperature of the 40-phase. In contrast to the 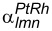 in Figure 8, the 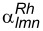 in this plot are biased due to surface segregation.

**Figure 10:**
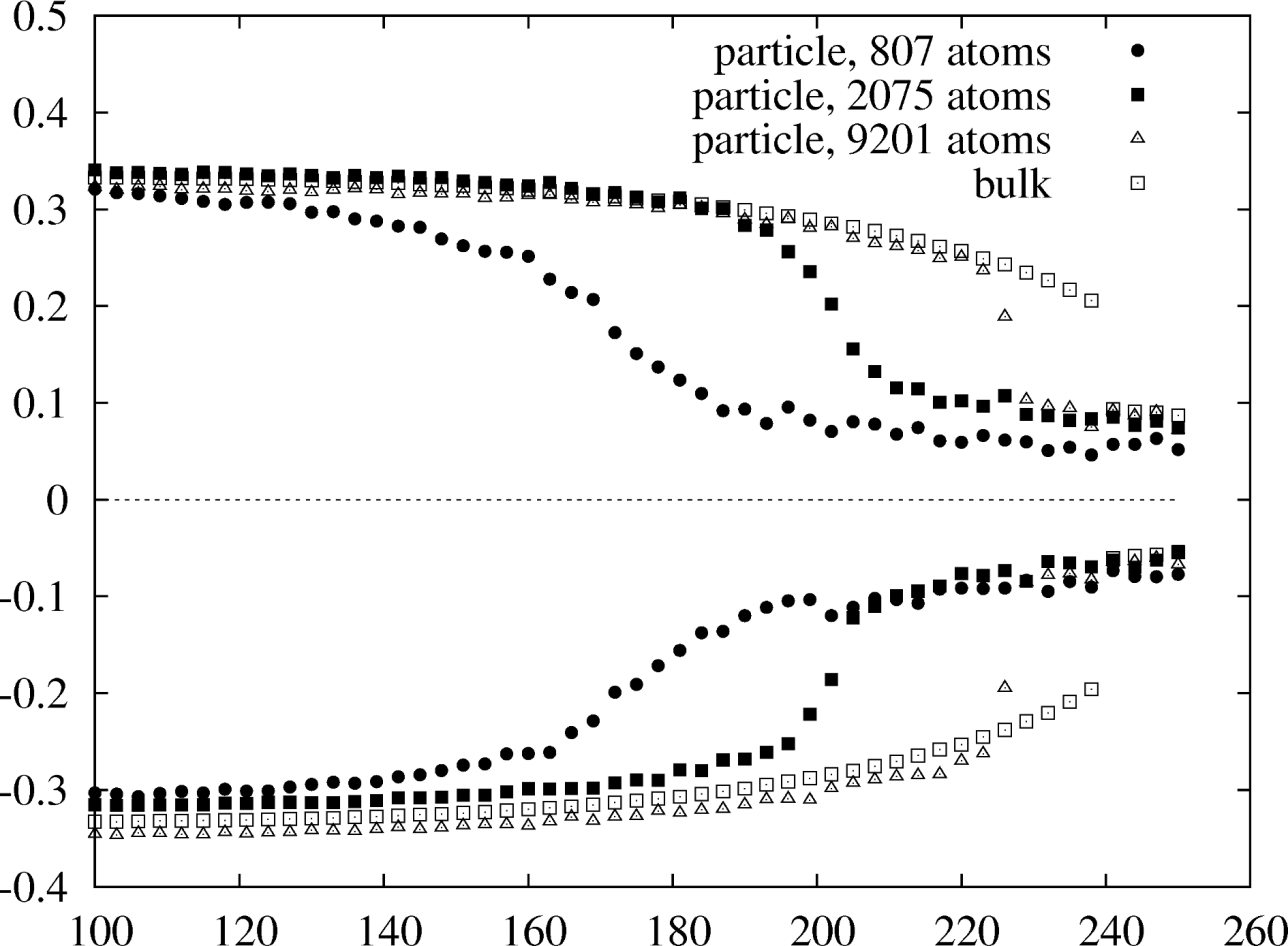
Comparison of the order parameters 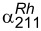 (lower points) and 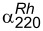 (upper points) constrained to a core region of 85 atoms. The Pt concentration is chosen such that for all particles the phase transition is observed at the maximum critical temperature of the 40-phase in the same way as in Figure 8. The ordering temperatures are the same as the ones obtained from the concentration-averaged order parameters in Figure 8.

We calculated the short-range order parameters for the three particle sizes using the definitions for 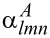, 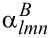 and 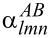, given in Equation 5 and Equation 6, which are equivalent for bulk alloys but not for particles, and core-restricted short-range order parameters for validation purposes. A full plot of all concentration-averaged WC-SRO parameters up to 8th neighbors for the large 7.8 nm particle is shown in Figure 6. In order to show how the particle size affects the order–disorder transition of the 40-phase we investigated two cases: First, we studied the order parameters at constant composition *x_Pt_* = 0.5 for the three particle sizes (Figure 7). Second, we adjusted the composition for each particle size to coincide with the maximum of the critical temperature, i.e., *x_Pt_* = 0.595, 0.66, 0.69 for the 7.8, 4.3 and 3.1 nm particles (Figure 8). For clarity only the two parameters for 6th and 8th neighbors that show the strongest variation at the transition (*lmn* = [222] and [400]), are shown in the plots. In both plots we look at the concentration-averaged order parameter 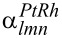. At 50 atom % platinum a transition can still be observed in the short-range order parameters at around 218 K for the 7.8 nm particle even though the ordered 40-phase in the core of the particle is now off-stoichiometric due to platinum segregation at the surface. For the 4.3 and 3.1 nm particle such a transition cannot be observed due to the enhanced surface segregation, which shifts the composition in the core of the particle into the two-phase region between *D*0_22_ and 40. This is an example of what can go wrong when bulk phase diagrams are used for the interpretation of the phase behavior of nanoparticles. In the bulk composition of the ordered 40-phase we do not find a single perfectly ordered phase for nanoparticles due to the concentration shift from surface segregation. In the second case at the compositions of maximum critical temperatures we find that for all particles an order–disorder transition can clearly be identified from the short-range order parameters (Figure 9). While for the bulk we see a jump in the order parameters, which indicates a first-order transition, the transition becomes continuous for the large particle with 9201 atoms. As the size of the particle is decreased the transition becomes increasingly smoothed out. In addition, we have also calculated the conventional short-range order parameters while restricting their evaluation to a small core region of the particles consisting of 85 atoms. In this case, the higher-order parameters are not defined anymore, but we can still evaluate them up to 4th neighbors (Figure 10). Comparison with the concentration-averaged order parameters in Figure 8 shows the same behavior in terms of the ordering temperatures obtained. The main difference is that the core-restricted parameters converge rather closely to the bulk values of the ordered phase, which is expected for a completely ordered core when surface atoms do not contribute.

For comparison, looking at the original non-concentration-averaged WC-SRO parameters 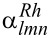 in Figure 9 we see that with decreasing size all of the order parameters 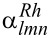 shift towards smaller values. This is a consequence of surface segregation. The segregation of platinum to the surface increases the probability of a Rhodium atom to see a Rhodium atom on one of its neighbor shells. Looking at the 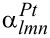 we would see a shift towards larger values. Taking the weighted average 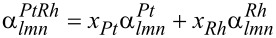 removes the surface segregation bias, such that the plot becomes symmetric about the zero line of the y-axis, as in Figure 8.

**Figure 11:**
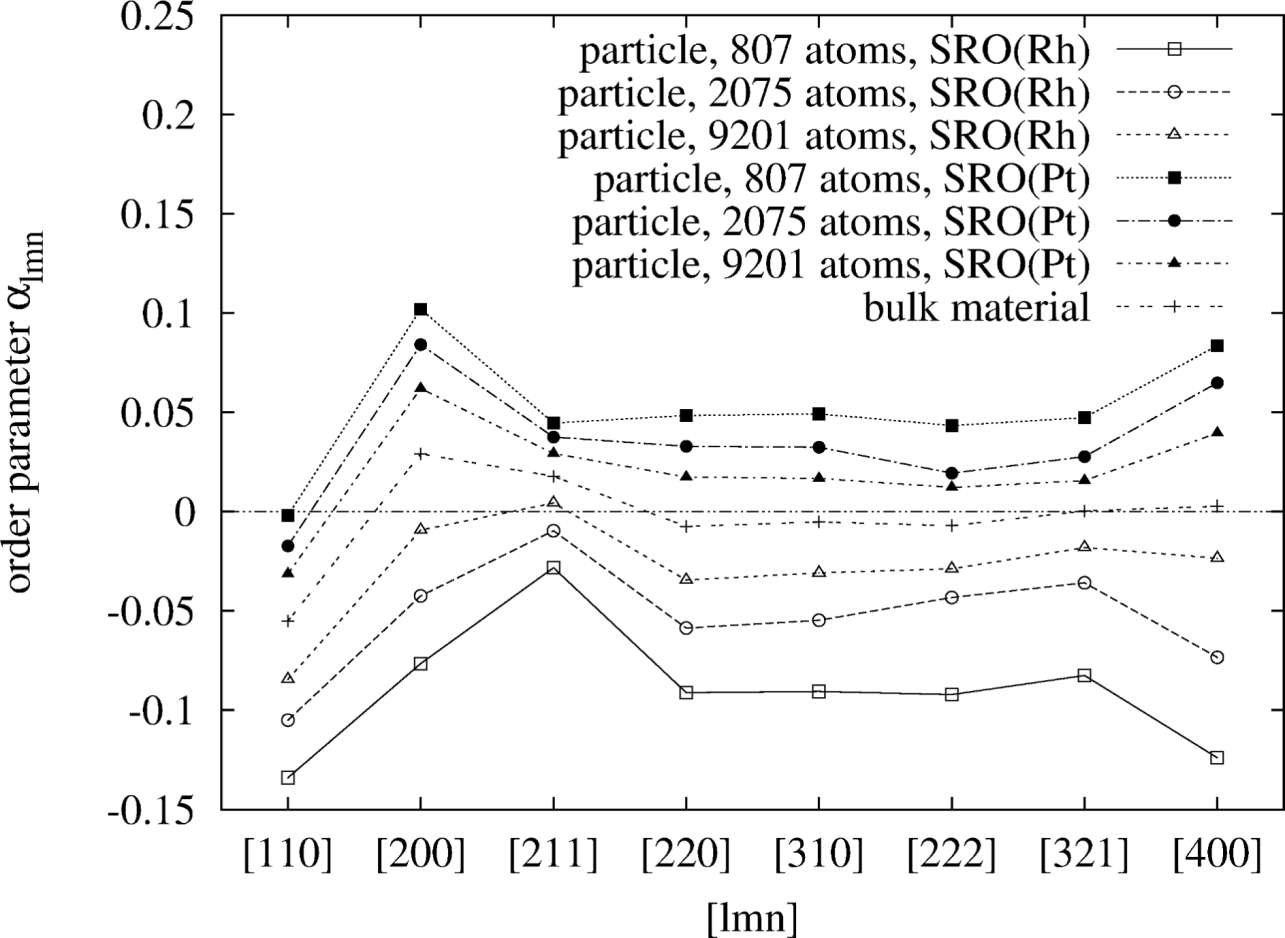
Comparison of the 8 WC-order parameters 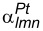 and 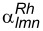 of three different particle sizes and bulk material at 923 K. The Pt concentration is 50 atom % in each case. The concentration of Pt on the surface layer is 64.9, 61.4, and 60.6 atom % in order of decreasing particle size.

**Figure 12:**
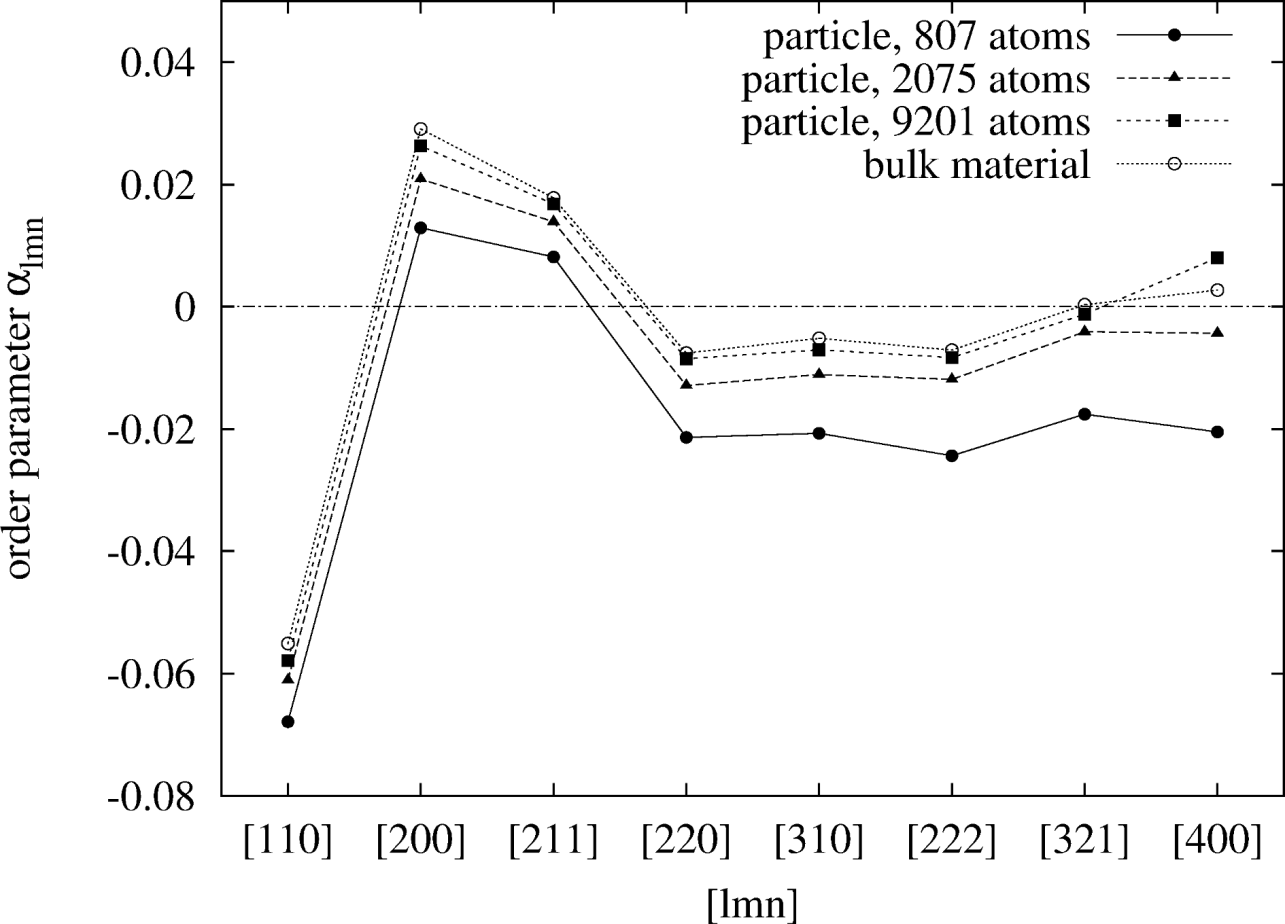
Comparison of the 8 averaged 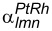 WC-order parameters of three different particle sizes and bulk material at 923 K. The Pt concentration is 50 atom % in each case. A decrease of 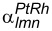 with decreasing particle size is clearly visible. The concentration of Pt on the surface layer is 64.9, 61.4, and 60.6 atom % in order of decreasing particle size.

Finally, the WC-SRO parameters at a temperature as high as 923 K were calculated (Figure 11) in order to be able to compare to experimental data [32–33] and to our former simulation for bulk Pt–Rh [4]. We find that the 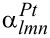 and 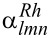 show significant shifts away from their bulk values. While all the 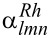 shift to smaller (more negative) values the 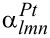 shift to larger values. This can be understood based on the same argument as outlined above. Surface segregation of platinum, which is still significant at these high temperatures, decreases the probability of finding platinum in the core. The effect becomes more pronounced with smaller particle sizes. Again, taking the concentration-weighted average 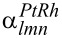 removes the shifts almost entirely (Figure 12), which shows that the short-range order in the core of the particles is essentially bulk-like. Only a very small shift towards more negative values remains. The fact that most of the effect of surface segregation on the short-range order is averaged out when concentration-averaged short-range order parameters are used, may be important for experiments in which this quantity can be measured.

## Conclusion

In this paper we discussed the size-dependency of the Pt–Rh phase diagram as obtained from lattice Monte Carlo simulations. Our results may serve as a paradigmatic test case for the change in phase transformation lines with a change in particle size. The broadening of the concentration range of ordered phases is concluded to be a consequence of the presence of the particle surface, which serves as a reservoir for excess atoms. When the surface is entirely covered with atoms of the segregating element the surface looses its function as a reservoir for excess atoms. This is consistent with a broadening of the *D*0_22_-phase concentration range, while little broadening is observed for the 40-phase. As a consequence, the *D*0_22_/40 two-phase region shrinks at the cost of the expanding *D*0_22_-phase, which shows a greater range of compositional stability because the surface reservoir is larger relative to the total number of atoms in the particle. It was also shown that the two-phase equilibrium in a particle may have complex morphologies (compare, e.g., the different shapes of the two-phase equilibria in Figure 4, particles 3, 4, 7 and 8). Finally, we found evidence that the formation of a two-phase core–shell equilibrium inside the particle may significantly affect surface segregation. In the present case of a core–shell equilibrium between the 40- and the *D*0_22_-phase the particle tends to increase the 40-phase core volume by diminishing the amount of segregated atoms and thus counterbalancing the driving force for segregation due to surface energy differences.

From the analysis of the Warren–Cowley short-range order parameters we found that the first-order bulk transition becomes continuous and increasingly smooth with decreasing particle size. When surface segregation of one element is present the ordered phase at 1:1 stoichiometry can only be stabilized by adjusting the global concentration in order to compensate for the lack of one species due to surface segregation. The Warren–Cowley order parameters 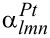 and 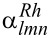 as classically defined by Cowley [30] show an increasing shift in their magnitude with decreasing particle size, which can almost entirely be removed by using concentration-averaged order parameters as defined by Hou et al. recently [24]. Analysis of the concentration-averaged short-range order parameters at 923 K showed that at these high temperatures the short-range order in the core of the particle is essentially bulk-like.
